# The dating of thrombus organization in cases of pulmonary embolism: an autopsy study

**DOI:** 10.1186/s12872-019-1219-8

**Published:** 2019-11-08

**Authors:** Gelsomina Mansueto, Dario Costa, Emanuele Capasso, Federica Varavallo, Giuseppina Brunitto, Rosanna Caserta, Salvatore Esposito, Massimo Niola, Celestino Sardu, Raffaele Marfella, Claudio Napoli, Mariano Paternoster

**Affiliations:** 1 Department of Advanced Biomedical Sciences, Legal Medicine Unit, University of Naples Federico II, Naples, Italy; 2 Department of Advanced Biomedical Sciences, Pathology Unit, University of Naples Federico II, Naples, Italy; 3 Clinical Department of Internal Medicine and Specialistics, U.O.C. Division of Clinical Immunology, Immunohematology, Transfusion Medicine and Transplant Immunology, University of Campania “Luigi Vanvitelli”, Naples, Italy; 4 Unit of Pathological Anatomy, Aversa Hospital, Caserta, Italy; 5 Department of Advanced Medical and Surgical Sciences, Università degli Studi della Campania “Luigi Vanvitelli”, Piazza Miraglia, 2 -, 80138 Naples, Italy

**Keywords:** Pulmonary embolism, Thrombus dating, Sudden unexpected death, Forensic autopsy, Histology

## Abstract

**Background:**

Pulmonary embolism (PE) is associated to high mortality rate worldwide. However, the diagnosis of PE often results inaccurate. Many cases of PE are incorrectly diagnosed or missed and they are often associated to sudden unexpected death (SUD). In forensic practice, it is important to establish the time of thrombus formation in order to determine the precise moment of death. The autopsy remains the gold standard method for the identification of death cause allowing the determination of discrepancies between clinical and autopsy diagnoses. The aim of our study was to verify the morphological and histological criteria of fatal cases of PE and evaluate the dating of thrombus formation considering 5 ranges of time.

**Methods:**

Pulmonary vessels sections were collected from January 2010 to December 2017. Sections of thrombus sampling were stained with hematoxylin and eosin. The content of infiltrated cells, fibroblasts and collagen fibers were scored using a semi-quantitative three-point scale of range values.

**Results:**

The 30 autopsies included 19 males (63.3%) and 11 females (36.7%) with an average age of 64.5 ± 12.3 years. The time intervals were as follows: early (≤1 h), recent (> 1 h to 24 h), recent-medium (> 24 h to 48 h), medium (> 48 h to 72 h) and old (> 72 h). In the first hour, we histologically observed the presence of platelet aggregation by immunofluorescence method for factor VIII and fibrinogen. The presence of lymphocytes has been identified from recent thrombus (> 1 h to 24 h) and the fibroblast cells were peripherally located in vascular tissue between 48 and 72 h, whereas they resulted central and copious after 72 h.

**Conclusions:**

After a macroscopic observation and a good sampling traditional histology, it is important to identify the time of thrombus formation. We identified histologically a range of time in the physiopathology of the thrombus (early, recent, recent-medium, medium, old), allowing to determine the dating of thrombus formation and the exact time of death.

**Clinical trial number:**

NCT03887819.

**Trial registration:**

The trial registry is Cliniclatrials.gov, with the unique identifying number NCT03887819. The date of registration was 03/23/2019 and it was “Retrospectively registered”.

## Background

Venous thromboembolism (VTE) is a common cardiovascular disease with high mortality rate worldwide [1]. It refers to a development of blood clots mostly into deep veins of lower extremities in absence of traumatic injury as deep vein thrombosis (DVT) that can detach from original sites and travel to the lung resulting in pulmonary embolism (PE). PE is influenced by genetic and environmental risk factors. There are several conditions such as surgery and trauma that impact on clinical consequencesof PE [2]. The diagnosis of PE result inaccurate, with many cases incorrectly diagnosed or missed, explaining its high percentage of mortality [3, 4]. Clinical presentation of PE range from those completely asymptomatic or with an insidious disease, to sudden unexpected death (SUD) [5, 6]. SUD may be defined as a natural and fatal event that occurs within 1 h of the beginning of symptoms in an apparently healthy subject or in those with a disease not particularly severe to cause an abrupt outcome [7]. Usually, it occurs in subject younger than 35 or 40 years. Since the diagnosis is challenging, epidemiological data regarding PE mortality remain limited. To date, it is estimated that PE is responsible for 100,000 annual deaths only in the United States and approximately 25–30% of patients had SUD as consequence of PE [1].

In forensic practice, PE is one of the major causes of SUD [8]. In case of vascular occlusion, it is essential to collect morphological macroscopic evaluation elements to distinguish a thrombus from an embolus or agonic coagulum [9, 10]. The autopsy may be useful for the determination of missed diagnoses in patients who died in the hospital and remain the gold standard method for the identification of death cause. Several studies have evaluated the discrepancies between incorrectly clinical diagnose of physicians and autopsy diagnoses [11–13]. So, it is of particular interest to establish the dating of transformation of thrombus and evaluate any professional error [13]. Considering medico-legal aspects it becomes fundamental to know if a pulmonary embolus is originated prior or subsequent to a traumatic event.

In this study, we critically reviewed the histological section of thrombus from 30 fatal cases of PE as confirmed by post-mortem examination and final diagnosis. The aim of this study was to evaluate the chronological transformation of the thrombus and to establish the time of death for these patients.

## Methods

### Patients and samples

Pulmonary vessels sections from 30 fatal cases of PE (25 cases of hospitalized patients and 5 cases of SUD) defined according to commonly accepted criteria [14] were routinely collected and obtained from January 2010 to December 2017 at the University of Naples Federico II. The pre-autopsy data were described in the Table 1. All the autopsies were performed according to the guidelines for autopsy from the Association for European Cardiovascular Pathology [10], and in all cases they were examined complete macroscopic autopsy with examination of the pulmonary arterial trunk by posterior approach. From the present study we excluded patients with diagnosis of systemic infection or underlying vasculitis.

**Table 1:** Clinical and demographic characteristics of pulmonary embolism cases

Patients	Age	Sex	Clinical history	Time between symptoms and death	Time between surgery and death	Time of hospitalization
1	48	M	No (SUD)	< 1 h	N/A	N/A
2	55	M	Surgery	< 1 h	72 h	96 h
3	55	M	Cardiomyopathy	< 1 h	N/A	96 h
4	78	F	Surgery	48 to 72 h	168 h	192 h
5	75	M	Cardiomyopathy	24 to 48 h	N/A	72 h
6	67	F	Cardiomyopathy	24 to 48 h	N/A	194 h
7	66	F	Surgery/Cardiomyopathy	48 to 72 h	114 h	162 h
8	76	F	Surgery/Cardiomyopathy	24 to 48 h	165 h	188 h
9	73	M	Surgery	< 1 h	136 h	163 h
10	54	F	Surgery	24 to 48 h	200 h	227 h
11	55	M	Surgery	< 1 h	178 h	220 h
12	65	F	Surgery	24 to 48 h	136 h	180 h
13	60	M	Surgery	< 1 h	203 h	228 h
14	70	M	Cardiomyopathy	after 72 h	N/A	72 h
15	48	M	No (SUD)	< 1 h	N/A	N/A
16	38	M	No (SUD)	< 1 h	N/A	N/A
17	65	M	Cardiomyopathy	24 to 48 h	N/A	24 h
18	44	F	Surgery	24 to 48 h	171 h	195 h
19	54	F	Surgery	48 to 72 h	201 h	237 h
20	76	M	Surgery	48 to 72 h	250 h	274 h
21	79	M	Cardiomyopathy	after 72 h	N/A	28 h
22	67	F	Cardiomyopathy	24 to 48 h	N/A	164 h
23	77	M	Cardiomyopathy	24 to 48 h	N/A	168 h
24	87	F	Surgery	48 to 72 h	89 h	113 h
25	49	M	No (SUD)	< 1 h	N/A	N/A
26	58	M	No (SUD)	< 1 h	N/A	N/A
27	69	F	Surgery/Cardiomyopathy	24 to 48 h	108 h	156 h
28	76	M	Surgery/Cardiomyopathy	< 1 h	107 h	179 h
29	78	M	Cardiomyopathy	48 to 72 h	N/A	72 h
30	72	M	Cardiomyopathy	< 1 h	N/A	72 h

### Histological evaluation

The original tissues samples were fixed in 10% neutral buffered formalin and embedded in paraffin blocks. Sections (4 μm thick) were stained with hematoxylin and eosin stain (H&E) for diagnosis. The immunohistochemistry for anti-LCA, anti-CD68, and anti-CD3 was performed to identify the inflammatory infiltrate. In addition, we performed an immunofluorescence method to identifying the deposition of factor VIII and fibrinogen. A Picro Sirius Red/Fast Green was used for differential staining of collagen during matrix production phase. All stained samples were examined under digital and light microscope. The content of CD3 positive lymphocytes and collagen fibers was scored using a semi-quantitative three-point scale of range values. For inflammatory cellular infiltration and fibrosis, we attributed score 0 for no increase, score 1, 2, or 3 for little, moderate, or high increase of cell content compared to adjacent tissue, respectively. For the extracellular matrix production, we attributed score 0 for absence of collagen production, score 1 and 2 for 10–40% and 40–80% collagen fibers content compared to adjacent normal tissue, respectively, and finally score 3 for wound matrix indistinguishable from adjacent normal tissue, as resumed in the Table 2.

**Table 2:** Histological score of inflammatory infiltrate and fibrosis

	0	1	2	3
Cellular infiltration	Absent	Little	Moderate	High
Fibrosis	Absent	10–40%	40–80%	> 80%

## Results

The 30 autopsies included 19 male (63.3%) and 11 female (36.7%). The time intervals were as follows: early (≤1 h), recent (> 1 h to 24 h), recent-medium (> 24 h to 48 h), medium (> 48 h to 72 h) and old (> 72 h) (Table 3). In 5 patients without clinical history of pre-existing pathologies the death occur in the first hour; thus they were considered cases of SUD. In these cases we do not observed cellular infiltration, fibrosis or neovascularization (Table 4). In the other cases, 10 patients have cardiomyopathy under pharmacological treatment, 11 were patients hospitalized for abdominal or fracture surgery and 4 patients with cardiomyopathy received surgery after head injury. However, in these 25 patients we had the following pathologies: hypertension (n 11, (44%)), diabetes (n 3, (12%)), peripheral angiopathy (n 6, (24%)), atrial fibrillation (n 3, (12%)), and cancers (n 2, (8%)). Regards the computed tomography exam, that were executed before the fatal death event, we reported a Mediastinal pulmonary artery (n 7 (23.3%)), Lobar pulmonary artery (n 5 (16.7%), Segmental pulmonary artery (n 15 (50%)), and Subsegmental pulmonary artery (n 3 (10%)) thrombus location. To date, from computed tomography exam we reported a clot volume of 2863.11 ± 853.08 mm^3^. The histological examination showed that 4 patients had a score 3 for cellular infiltrate and a score 2 for fibrosis platelet aggregation below the vascular endothelium with fibrin accumulation, small or absent erythrocyte infiltrate, absent or rare lympho-monocytic inflammatory elements. The vessels of small and medium were involved, mainly from the main branches of the pulmonary vessels. In2 patients we observed a score 3 for cellular infiltrate and 1 for fibrosis. In 10 cases (5 heart patients, 2 cardiac patients undergoing surgery and 3 surgeries), we observed an increase (score 2) in the lymphocyte-monocytic cellular component (CD3+), but also in the histiocytic component (CD68+), recruited as a consequence of initial erythrocyte degradation. Moreover, 7 patients have only a slight or absent cellular infiltration (score 1/0) without fibrosis (score 0). Finally, 2 cardiopathic subjects with symptomatic onset 72 h before death showed fibrous substitution (score 3) in the absence of inflammatory share (score 0) with or without revascularization.

**Table 3:** Score related to dating thrombus formation

Score	Early (1 h)	Recent (1 h–24 h)	Recent-medium (24 h–48 h)	Medium (48 h–72 h)	Old (> 72 h)
Inflammatory cells	0	1	1/2	2/3	0/1
Fibrosis	0	0	1	2	3

**Table 4:** Score of histological observation with optical microscopy

Patients	T-cell infiltration (CD3+)	Fibrosis	Neovascularization
1	0	0	0
2	1	0	0
3	0	0	0
4	3	2	0
5	2	0	0
6	2	0	0
7	3	2	0
8	2	0	0
9	1	0	0
10	2	1	0
11	1	0	0
12	2	1	0
13	1	0	0
14	0	3	+
15	0	0	0
16	0	0	0
17	2	1	0
18	2	1	0
19	3	1	0
20	3	1	0
21	0	3	+
22	2	1	0
23	2	1	0
24	3	2	0
25	0	0	0
26	0	0	0
27	2	1	0
28	1	0	0
29	3	2	0
30	0	0	0

**Fig. 1:**
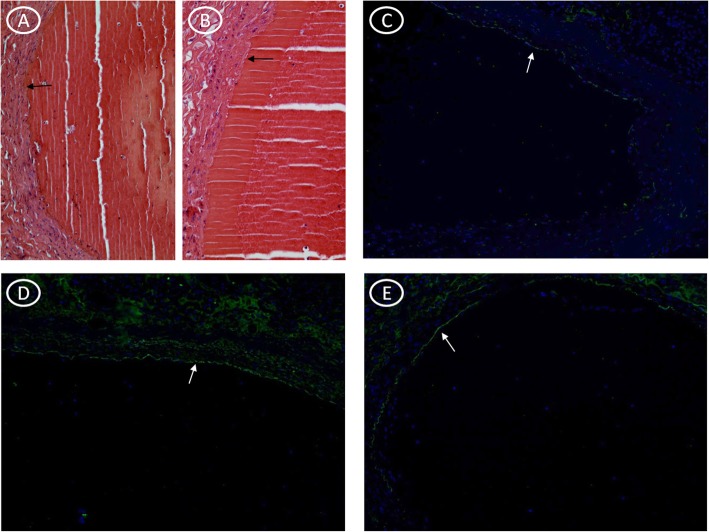
Thrombus formation in the first hour in SUD. **a)** H&E staining showing the thrombus overview of platelet aggregation below the vascular wall (5x magnification); **b)** H&E staining showing the thrombus overview of platelet aggregation below the vascular wall (10x magnification); **c)** Immunofluorescence indicating the accumulation of factor VIII in the vascular wall (white arrow); **d-e)** Immunofluorescence indicated fibrinogen amount in the vascular wall (white arrows)

**Fig. 2:**
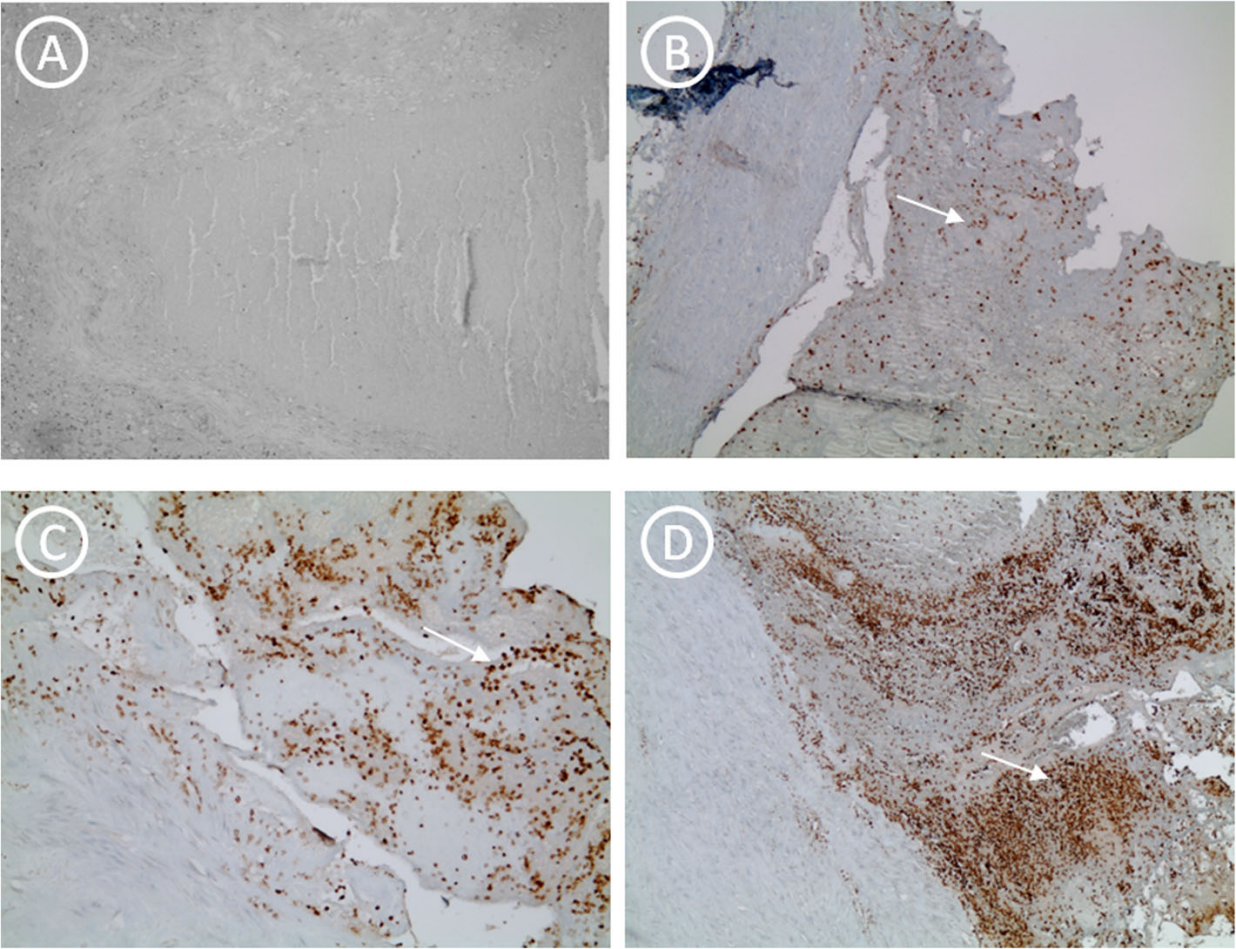
Immunohistochemistry for anti-CD3 antibody at different times. **a)** Negative immunohistochemistry for anti-CD3 in a case of SUD (10x magnification). The white arrow indicates the T lymphocytes at different time **b)** > 1 h to 24 h; **c)** > 24 h to 48 h; **d)** > 48 h to 72 h in different cases of PE death

**Fig. 3:**
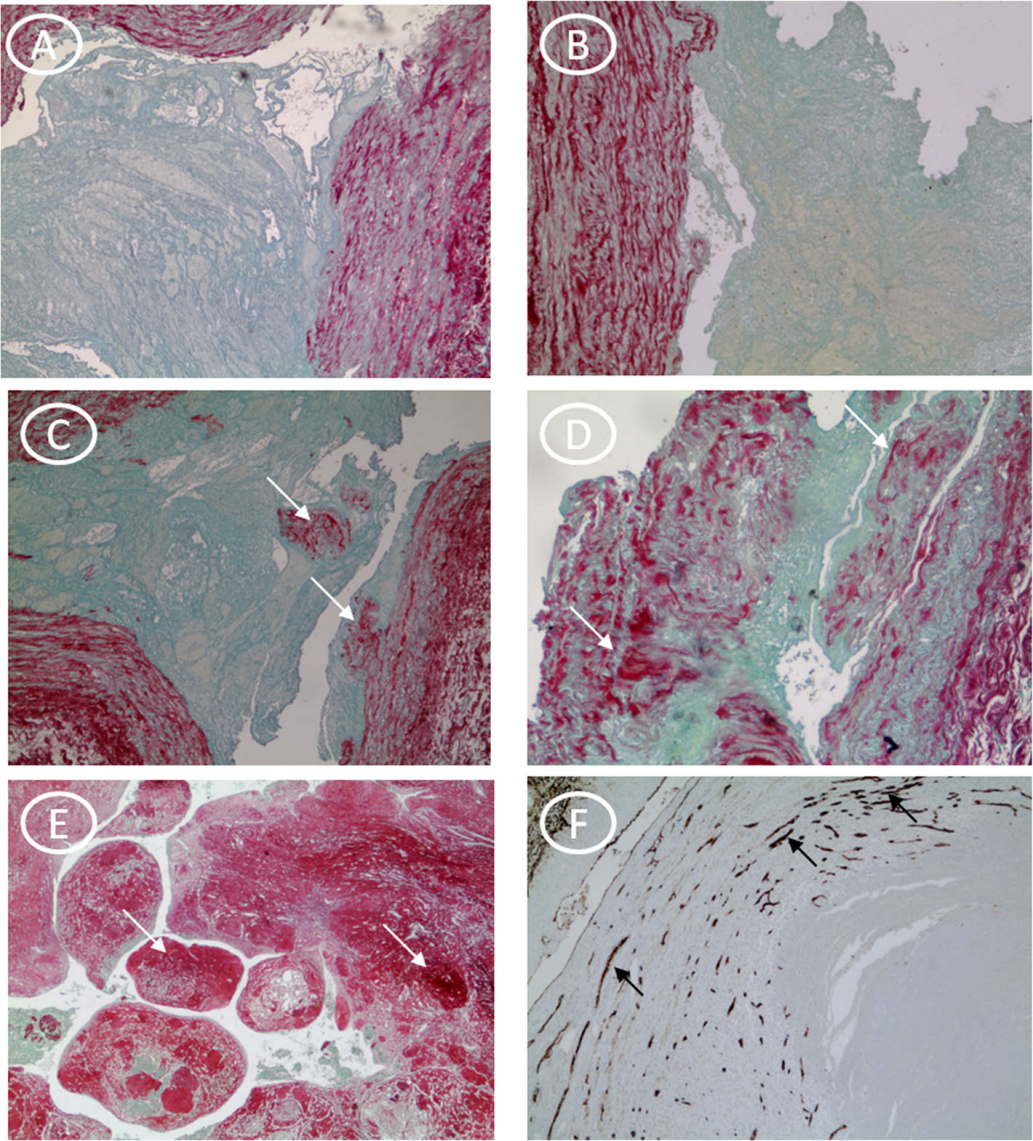
Fibrosis score atdifferent times and revascularization. **a)** In the first hour and **b)** up to 24 h’ absence of fibrosis with absence of red signal. In the following hours **c)** > 24 h to 48 h, **d)** > 48 h to 72 h, **e)** > 72 h there is an increase in the signal (PricoSirius Red/Fast Green stain; 10x magnification). **f)** The black arrow indicates revascularization in the old thrombus after 72 h (Immunohistochemistry for anti-CD31 antibody; × 10 magnification)

In the first hour, we histologically observed the presence of platelet aggregation with few or no erythrocytes (Fig. 1a,b) and we defined it as precocious valuated by immunofluorescence method for factor VIII **(**Fig. 1c**)** and fibrinogen (**Fig.** **1****d,e).** The gray thrombus is made of platelets and fibrin, while red blood cells in the fibrin network characterize the red thrombus. Immunohistochemistry stain evidenced no presence of lymphocyte (T cell) infiltration within an hour (Fig. 2a). The presence of lymphocytes has been identified from recent thrombus (> 1 h to 24 h) (Fig. 2b). In the recent-medium thrombus, we observed the increased of the inflammatory cells **(**Fig. 2c**),** but we observed the initial degradation with histiocytic and fibroblasts progressive recruitment up to the medium thrombus (Fig. 2d). Lysis of leukocytes involves the release of enzymes with subsequent digestion and destruction of erythrocytes and platelets with prevalent fibrin and cellular debris (homogenization). The fibroblast cells and fibrosis were peripherally located in vascular tissuein the early hours and up to the third day (Fig. 3a,b,c,d) whereas they resulted central and copious in the fourth day (Fig. 3e,f). Finally, in the old thrombus, we have observed a proliferation of endothelial cells, increase of fibrosis and recanalization phenomena (Fig. 3f).

## Discussion

The major finding of this study was the determination of thrombus formation and its timely evolution in correlation to the death event. In particular, we identified platelet activation through factor VIII and fibrinogen during the first hour. Secondly, we evaluated the inflammatory infiltrate and the evolution of the fibrotic state from 1th to 72th hour. In forensic practice, PE is one of the major causes of SUD [13, 15, 16]. The accuracy of ante-mortem diagnosis is within the range of just 10–30% [17], representing one of the most frequent missed diagnosis in SUD. This provides a setting for malpractice claim. When in autopsy was observed vascular occlusion, it is essential to collect morphological macroscopic evaluation elements to distinguish a thrombus from an embolus or agonic coagulum. Macroscopically, it is important to note that the native thrombus adheres to the walls of the vessel in correspondence with an endothelial lesion, which is then examined by microscopy. Moreover, the arterial thrombus is generally occluding, adheres to a parietal lesion in general atherosclerotic, is gray-white in color and friable and has the classic striped appearance [1]. On other hand, the venous thrombus is red and creates an endoluminal mold [1]. Instead, thrombus-embolus starts from a different vascular territory and is formed in the presence of an intact vessel. In addition, it’s important to identify the time of thrombus formation because it correlates with the time of death; thus, it is possible to evaluate with high probability if the clinical treatment has been corrected [12]. Several studies have evaluated the discrepancies between clinical and autopsy diagnoses, demonstrating that the use of different imaging techniques seldom contribute directly to inaccurate or missed diagnoses [18–20]. Despite progress in imaging techniques and therapeutic treatment, the importance of autopsy remains crucial to elucidate the cause of death. However, there are not forensic histological guidelines to determine the thrombus dating and its association to the death cause. An interesting study have reported a dating setting a range from 1 to 7 days, from 2 to 8 weeks and over 2 months [21]. This study introduces a methodological approach that combines clinical data derived from autopsy and histological analysis; so, it appears to be the first valid approach for the determination of thrombus formation through histological criteria. A recent case report highlighted that although pathology skills are mandatory for an accurate chronological evaluation of fatal PE, histopathology and immunohistochemistry play both a crucial role in the evaluation of PE to the specific case [22]. Therefore, our experience in forensic thanatology [23, 24] reinforces the concept that microscopic observation is at the basis of a correct diagnosis and able to provide informations also for forensic practice and in the specific case in the age-old question of dating [13]. In cases of SUD, the estimate of small time intervals in thrombus formation is difficult but possible. Indeed, in the absence of anamnesis due to an arrhythmic events or other causes of cardiac death, they have been initially labeled as thrombus-embolic events. For the remaining cases, the classification in a time was fundamental to validate the diagnostic process and the treatment of patients with the aim of identifying a causal link that led to the death event. Moreover, we can compare the formation of thrombus and its resolution to wound healing. In both of these processes, in fact, the inflammatory elements, the bioactive molecules, such as cytokines and growth factors, and the remodeling of the matrix play an important role [25–27]. Indeed, all these multiple cellular and extracellular pathways favor a pro-thrombotic status, that might be reversed by opposite anti-thrombotic mechanisms causing thrombus resolution [1]. On the other hand, the loss of these anti-thrombotic protective mechanisms might consequently evolve towards worse prognosis and SUD events in patients with PE [3–8]. To date, thrombi causing pulmonary embolism are rich in fibrin and trapped red blood cells, and are referred to as red clots [28]. Conversely the pro-thrombotic status is enhanced by over activity or abundance of proteins that promote coagulation and/or decreased abundance of proteins that inhibit coagulation [29]. In this setting, the coagulation cascade represented by three pathways as the extrinsic pathway (tissue factor and factor VIIa, which is the primary activator of the cascade), the intrinsic pathway (factor XIIa, factor XIa, factor IXa and factor VIIIa, which amplifies the cascade), and the common pathway (factor Xa, factor Va and thrombin, which generates thrombin and fibrin), plays a relevant role in thrombotic processes [30]. However, we might speculate that, in the early phase of thrombus formation a specific anti-thrombotic therapy might inhibit a specific target pathway of the coagulation cascade [31]. This therapeutic effect might consequently impact on ongoing thrombotic process [31], and this might consequently ameliorate clinical outcomes in patients with EP. We are aware that this retrospective study has major limitations represented by the small number of autopsies included and the inter-operator sampling variability but our considerations may be useful for successive studies aimed to evaluated the dating of thrombus formation. Moreover, the acquired and inherited thrombophilic risk factors for PE is not considered. In the present research we have identified by histological analysis a timing in the physiopathology of the thrombus (early, recent, recent-medium, medium, old). Since often the antigenicity of tissues and cells is lost with post-mortal phenomena, the risk of detect false positives or negatives to immunohistochemistry analysis might increase. Therefore, we believe that the traditional histology after a macroscopic observation and after a good sampling is important to identify the time of thrombus formation. The immunohistochemically and immunofluorescence methods might only confirm what morphologically the pathologist observes.

## Conclusions

In the present study we identified by histological analysis a timing in the physiopathology of the thrombus formation as early, recent, recent-medium, medium, and old, allowing to determine the exact time of death. However, during the 1th hour of thrombosis we reported a first thrombotic process caused by platelet activation through factor VIII and fibrinogen. Consequently, a second phase and timing of thrombosis is caused by the inflammatory infiltrate and the evolution of the fibrotic state from 1th to 72th hour. In our opinion, a deeper knowledge of the different phases of thrombus formation and of its link with time of death might open new investigations to develop new biomarkers for the early identification and the monitoring of patients with EP and higher risk of death. Conversely, the identification of different timing and pathological processes during thrombosis and EP events might open a new scenario regards the opportunity to practice a prophylactic anticoagulation, according to risk factors, in patients with thrombi formed prior or following a traumatic event. Therefore, we might speculate that the time to thrombus formation and death might change the current clinical practice. Indeed, this might cause a possible modification of the current antithrombotic strategy by the selective therapeutic modulation and block of the factor VIII and fibrinogen during first phase of thrombosis, and subsequently of the inflammatory infiltrate during the second phase of thrombosis. Finally, this might find a greater application in the current clinical practice to ameliorate clinical outcomes and to reduce deaths in patients with EP.

## Data Availability

The datasets used and/or analyzed during the current study are available from the corresponding author on reasonable request.

## Abbreviations

CD3+: lymphocyte-monocytic cellular component
CD68+: histiocytic component
DVT: Deep vein thrombosis
H&E: Eosin stain
PE: Pulmonary embolism
SUD: Sudden unexpected death
T cell: Lymphocyte
VTE: Venous thromboembolism
